# Neural pathways in nutrient sensing and insulin signaling

**DOI:** 10.3389/fphys.2022.1002183

**Published:** 2022-11-11

**Authors:** Anuradha Ratnaparkhi, Jyothish Sudhakaran

**Affiliations:** ^1^ Department of Developmental Biology, MACS-Agharkar Research Institute, Pune, India; ^2^ Savitribai Phule Pune University, Pune, India

**Keywords:** *Drosophila*, neurons and nutrient sensing, insulin-like peptides, IPCs, metabolism

## Abstract

Nutrient sensing and metabolic homeostasis play an important role in the proper growth and development of an organism, and also in the energy intensive process of reproduction. Signals in response to nutritional and metabolic status is received and integrated by the brain to ensure homeostasis. In *Drosophila*, the fat body is one of the key organs involved in energy and nutrient sensing, storage and utilization. It also relays the nutritional status of the animal to the brain, activating specific circuits which modulate the synthesis and release of insulin-like peptides to regulate metabolism. Here, we review the molecular and cellular mechanisms involved in nutrient sensing with an emphasis on the neural pathways that modulate this process and discuss some of the open questions that need to be addressed.

## 1 Introduction: Insulin signaling

Nutrition is critical for growth and development of an organism. Nutrients in the form of glucose, amino acids and lipids activate specific signaling pathways that ultimately lead to generation of energy through synthesis of energy rich molecules like ATP essential for development, growth, reproduction and survival of the organism. Insulin signaling is an evolutionarily conserved pathway that integrates growth and nutrition. In vertebrates, insulin regulates the level of circulating glucose and thereby metabolism, while insulin-like growth factors regulate growth (Nakae et al., 2001). Insects have varying number of insulin-like peptides (ILPs) that function as both, regulators of glucose as well as growth factors (Brogiolo et al., 2001; Rulifson et al., 2002; Geminard et al., 2006; Slaidina et al., 2009; Okamoto et al., 2009; Nassel and Broeck., 2016).

The brain is one of the key organs involved in regulating circulating glucose levels. In vertebrates, glucose sensitive neurons in the hypothalamus, both excitatory and inhibitory, are involved in this process (Anand et al., 1964; Oomura et al., 1969). Insects including *Drosophila*, have multiple insulin-like peptides some of which are expressed by a cluster of median neurosecretory cells in the brain-also referred to as insulin producing cells or IPCs (Wu and Brown., 2006; Nassel and Broeck., 2016; Brogiolo et al., 2001; Ikeya et al., 2002; Rulifson et al., 2002). The nutritional and metabolic status of the organism is relayed to the IPCs, enabling them to respond appropriately.

Insulin-like peptides and elements of the insulin signaling pathway are conserved across phyla (Wu and Brown., 2006; Nassel and Broeck., 2016). The signaling pathway regulates and influences a diverse set of developmental and physiological processes including aging and sleep (Tartar et al., 2001; Broughton and Patridge., 2009; Metaxakis et al., 2014; Palermo et al., 2022; Yamaguchi et al., 2022). Unlike vertebrates which have a single insulin peptide and a family of insulin receptors (IR) and insulin-like growth factor receptors (IGFRs), *Drosophila* has 8 Insulin-like peptides (ILPs) and a single receptor. While DILPs 1-7 signal *via* the single Insulin receptor (InR), DILP8 signals through LGR-a G-protein coupled receptor (GPCR) with leucine rich repeats (Brogiolo et al., 2001; Gronke et al., 2010; Colombani et al., 2015; Garelli et al., 2015; Nässel and Vanden Broeck, 2016).

DILPs show spatio-temporal specificity in their expression pattern: DILPS 2,3, and 5 are produced and secreted by a set of 14 the median neurosecretory cells (MNCs) or insulin producing cells (IPCs) located dorsally in the larval and adult brain. These cells also express *dilp1* mRNA during larval stages (Rulifson et al., 2002) However, a more detailed study by Nassël has shown that DILP1 expression in IPCs begins in early pupae and continues till about 7 days post-eclosion in the young adults (Liu et al., 2016). DILP4 is expressed in the midgut and muscles in the embryo; DILP6 is expressed by the fat body and glia during the larval and pupal stages (Brogiolo et al., 2001; Ikeya et al., 2002; Rulifson et al., 2002; Salidina et al., 2009; Nassel et al., 2015; Okamoto and Nishimura., 2015; Liu et al., 2016).

In mammals, insulin/IGF molecules are synthesized as a pre-pro-peptide with a signal sequence followed by B, C and A peptides. In case of insulin, the C peptide is cleaved and, individual A and B peptides are linked together by disulphide bridges and packaged into secretory granules (Weiss M, 2009). In the case of IGF, the C peptide is small and does not undergo cleavage, resulting in a continuous peptide.

Amino acid alignment of all the ILPs in different species of *Drosophila* shows that the region of the A and B peptides is more conserved than the signal sequence and the C-peptide. Further, the cleavage sites for C peptide and the cysteines involved in the formation of the disulphide linkages between A and B peptide is also conserved. Interestingly, in case of DILP6, the C peptide is short as in IGF suggesting that it may function more as a growth factor (Grönke et al., 2010).

In summary unlike mammals where insulin and IGF peptides signal through independent receptors, in *Drosophila*, a single common receptor functions downstream of different ILPs. The signaling pathway involves phosphorylation of a single insulin receptor substrate (IRS) called chico–the mammalian homolog of IRS1-4, which further activates phosphoinositide-3-kinase (PI3K), phosphoinositide dependent kinase1, and protein kinase B (Akt1) to repress the transcription factor dFOXO (Barthel et al., 2005; Grewal, 2009). Inactivation of insulin signaling, leads to activation of dFOXO, which regulates transcript levels of 4EBP- a translational repressor and direct target of dFOXO (Barthel et al., 2005; Grewal, 2009). Thus, mRNA levels of 4EBP are upregulated in mutants of *ilps 2,3,5*.

Interestingly, 4EBP levels remain unchanged in single mutants of *dilp 2,5*; in *dilp3* mutants, 4EBP levels are elevated in the head but not body, indicating a degree of functional redundancy between DILPs secreted by the IPCs. Studies also suggest that at least amongst the DILPs secreted by the IPCs, there is preferential response to specific nutrients: DILP2 regulates circulating sugar levels; DILP5 and DILP3 are associated with protein and lipid metabolism respectively (Columbani et al., 2003; Géminard et al., 2009; Rajan and Perrimon, 2012; Koyama and Mirth, 2016). Thus, IPCs are central to sensing nutritional status and crucial for metabolic regulation. In this review we examine cellular and molecular mechanisms of nutrient sensing in *Drosophila* and the neuronal circuits involved therein.

## 2 Nutrient sensing mechanisms in the fat body

Nutrient sensing strategies at the level of individual cells leads to activation of the conserved TOR signaling pathway, a central player in coupling nutrient availability and growth (Hafen E, 2004; Carroll and Dunlop, 2017; Kim and Guan., 2019). However, at the organismal level, the process involves humoral signals emanating from nutrient “sensing” centres to ensure co-ordinated growth and regulated metabolism (Britton and Edgar., 1998). Since growth in *Drosophila* is restricted to the larval stages, changes in nutrient conditions during this stage affects organism size while in the adult, leads to altered metabolism. TOR signals through two different complexes namely TORC1 and TORC2. TORC1 is sensitive and responds to changes in amino acids while TORC2 is needed for cell growth and regulation of cellular cytoskeleton.

**FIGURE 1:**
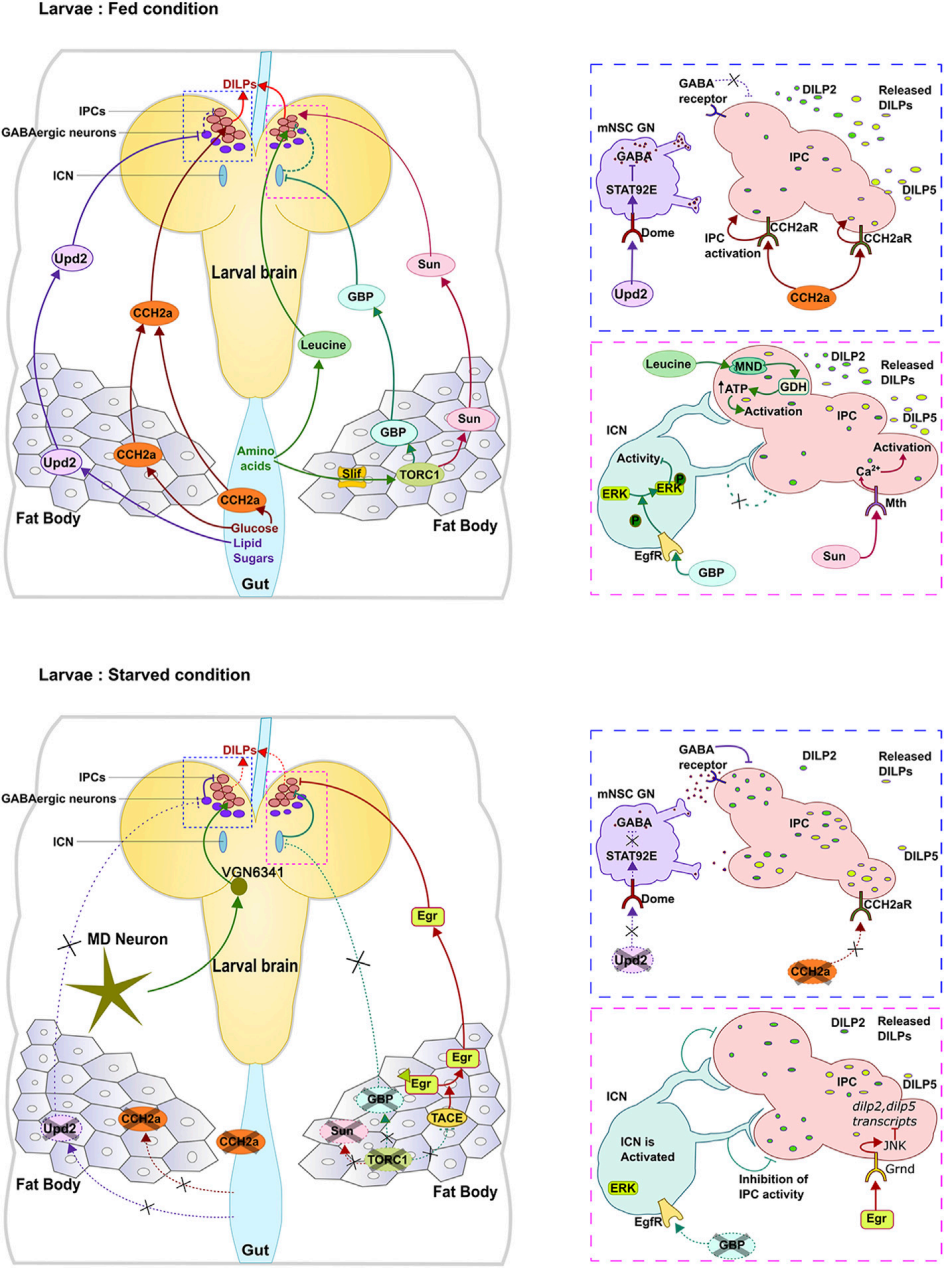
Regulation of IPCs in the larval brain under fed and starved conditions. Different nutrients lead to release of different signaling molecules from the fat body and gut to regulate DILP secretion.

In the larva, the fat body is one of the major nutrient sensing organs that stores lipids, carbohydrates and proteins which are mobilized for use under conditions of nutrient deprivation or during tissue remodeling in the pupal stages. Consistent with this role, knock-down or inhibiting elements of TOR signaling in the fat-body leads to delayed growth and defects in development. Multiple nutrient sensing mechanisms operate in the fat body which eventually signal to the IPCs to modulate release of DILPs (Figure 1). Amino acid transporters Slimfast (Slif) and Minidiscs (Mnd) are expressed in the fat body and serve as protein ‘sensors’ by responding to amino acids arginine and leucine respectively (Jayakumar et al., 2018; Martin et al., 2000; Reynolds et al., 2009). Slif functions upstream of TOR signaling in the fat body leading to release of DILP2 from the IPCs (Columbani et al., 2003; Géminard et al., 2009). Mnd on the other hand, is also expressed by the IPCs and can directly regulate rapid release of DILPs 2 and 5 in response to systemic leucine (Manière et al., 2016). Thus, in the larva, secretion of DILPs 2,5 is sensitive to protein levels and is regulated by TOR signalling in the fat body.

Carbohydrates and sugars constitute a critical source of energy for cells. The mechanism of intracellular sensing of sugars is conserved and involves two basic helix-loop-helix transcription factors Mondo and Max like protein X (Mlx). Unlike vertebrates which have two paralogs of Mondo, *Drosophila* has a single Mondo gene which, in the larva, is highly expressed in the fatbody, midgut and malphigian tubules (Havula and Hietakangas., 2012; Havula and Hietakangas., 2018). The Mondo-Mlx complex regulates transcription of a large number of genes involved in glycolysis, the pentose phosphate pathway and lipid synthesis (Mattila et al., 2015). In the larva, circulating glucose is taken up by the fat body and converted into trehalose–a non-reducing disaccharide, an energy source used by various tissues in insects (Becker et al., 1996; Shukla et al., 2015). Glucose also gets converted into glycogen and triacylglycerol (TAG) for storage. Ablation of IPCs increases the levels of circulating glucose, trehalose and TAGS indicating a role for the DILPS 1, 2,3, and 5 in carbohydrate metabolism (Brogiolo et al., 2001; Rulifson et al., 2002). In the larva, IPCs sense and respond to circulating trehalose indirectly *via* release of the adipokinetic hormone (Akh), the functional homolog of glucagon. Circulating trehalose triggers release of Akh by corpora cardiaca (CC) cells of the ring gland, which then signals to the IPCs leading to release of DILP3 (Kim and Neufeld. 2015). Like trehalose, glucose sensing by the IPCs during larval stages is indirect and dependent on signals released by the gut and the fat body (Sano et al., 2015). In the adult however, glucose can be sensed directly by the IPCs leading to release of DILP peptides through depolarization of ATP dependent potassium channels (_ATP_K^+^; Kim and Rulifson., 2004). In summary, the fat body employs different molecular strategies to sense nutrients. The signals that these mechanisms elicit is discussed below.

## 3 Humoral signals from the fat body

In *Drosophila*, the IPCs integrate nutritional information to regulate DILP release thus coupling nutritional status with growth. The IPCs are unique in that, they appear to be genetically programmed to couple nutrition and secretion. Thus, ectopically expressed secretory proteins that are not a target of the nutrient signalling also tend to accumulate under nutrient deprivation (Géminard et al., 2009).

As stated above, the fat body is a major nutrient ‘sensor’. In the larva, the fat body is present as a contiguous tissue of polyploid cells surrounding the central gut on either side (Aguila et al., 2007; Aguila et al., 2013). This positioning of the fat body allows it to be in close proximity to all organs including the gut, brain, muscle and gonad-a feature essential for efficient inter-organ signalling. In contrast, the adult fat body is discontinuous, located in specific regions along the body axis. The cells are diploid, derived from the larval body wall and adepithelial cells of the imaginal disc (Hoshizaki et al., 1995; Musselman and Künhlein, 2018).

That signaling exists between the fat body and the IPCs was first established through *ex-vivo* and fat body-larval brain co-culture experiments. These studies showed that DILP levels in the brain is altered when co-cultured with fat body derived from a starved or fed larvae indicating presence of non-autonomous signals that convey nutritional status to the brain (Britton and Edgar, 1998; Géminard et al., 2009).

It is now known that multiple signals are released by the fat body, each in response to different sets of nutrients (Figure 1). The cytokine Upd2, one of the earliest signalling molecules to be identified, is released in response to fats and sugars in both larval and adult stages. Flies mutant for *upd2*, feed normally yet exhibit high levels of circulating sugars and store less fat. Conversely, transcript levels of *upd2* are more than two-fold upregulated in animals fed with a high fat and high sugar diet indicating sensitivity to lipids and sugars. Further, DILPs 2 and 5 are seen to accumulate in the IPCs of these mutants at both, larval and adult stages, indicating that Upd2 likely signals the “fed” state (Rajan and Perrimon., 2012). Interestingly, expression levels of *upd2* are unaffected in animals with reduced slif expression indicating lack of sensitivity to amino acids.

The other glucose responsive signal released by the larval fat body and gut is the peptide hormone CCHamide2a whose receptor is present on the IPCs and directly activated by the ligand. In the absence of CCHamide2a, DILP2 accumulates in the IPCs accompanied by a decrease in levels of DILP5. Curiously, while inhibition of TOR signalling in the fatbody downregulates expression of this peptide, feeding animals amino acids including leucine, isoleucine has no effect on ligand expression. Thus, the CCH2a/CCH2a-R signalling system appears to be primarily responsive to glucose and directly connects the peripheral “sensor” to the “central” sensor of nutrition (Sano et al., 2015).

Proteins sensed by the fat body through activation of TOR signalling leads to release of signalling ligands. High protein levels trigger release of EGF-like growth blocking peptides (GBPs) 1 and 2 from the fat body while conditions of low protein activate signalling *via* Eiger-an adipokine belonging to the TNFα family (Agarwal et al., 2016; Koyama and Mirth., 2016).

Expression of *gbp1,2* mRNA in the larval fat body is regulated by TOR signalling and is responsive to dietary protein: animals fed with high protein show elevated mRNA expression of these genes. GBPs act non-autonomously to regulate expression and secretion of DILPS from the IPCs. Interestingly, individual knockdown of each of these genes leads to increased accumulation of DILP5 but not DILP2. However, downregulation of both *gbp* genes using RNAi results in accumulation of both DILPs which is also observed in double mutants of *gbp1* and *gbp2*. Curiously, the double mutants fail to show a correlation between protein and mRNA levels: *dilp2* mRNA levels are elevated while *dilp5* levels are diminished in the mutants (Koyama and Mirth., 2016).

Eiger, on the other hand, is a transmembrane protein secreted under low protein conditions. The ectodomain of the protein is cleaved by TACE (TNFα converting enzyme-a metalloprotease) and released into the hemolymph. The cleaved region, binds to its receptor Grindelwald to activate JNK signalling in the IPCs. Expression TACE in the fat body is regulated by TOR signalling (Agarwal et al., 2016) thus making the signalling process sensitive to protein levels.

In addition to GBPs and Eiger, the fat body also secretes peptides SunA and SunB which are derived from the gene *stunted*. Expression and secretion of Sun peptides is sensitive to proteins: lowering TORC1 activity in the fat body leads to a reduction in circulating Sun levels. Furthermore, overexpression of Sun can partially rescue the growth phenotype in *slif* mutants (Delanoue et al., 2016). Sun peptides signal *via* Methuselah–a GPCR present on the IPCs leading to changes in intracellular calcium and DILP secretion (Cvejic et al., 2004; Delanoue et al., 2016). Thus, multiple signals are released to convey “protein levels” to the IPCs. The same appears to be true for sugars as well indicating redundancy in the ‘sensing’ process. Furthermore, the mechanism by which these signals regulate IPC function are also distinct (Table 1): While some act directly, other act through neural intermediates to regulate IPC function.

**TABLE 1:** Molecules associated with nutrient sensing and their effect on IPC derived ILPs.

Mutant genotype	Nutrients sensed	Dilp2	Dilp5	Dilp3 levels	References
Upd2	Glucose and Lipids	DILP2 accumulates in IPCs	DILP5 accumulates in IPCs	Not tested	Rajan and Perrimon. (2012)
CCHamide	Glucose and fat	DILP2 accumulates in IPCs. No change in mRNA levels	Decrease in DILP5 in IPCs Dilp5 mRNA levels reduced	No change	Sano et al. (2015)
Stunted	Responds to starvation	DILP2 accumulation	Not known	Not known	Delanoue et al. (2016)
Slif/TOR inhibition	Amino acids	No change in transcription of *dilps*. Reduced secretion of DILP2 : the protein accumulates in IPCs	Not known	Not known	Columbani et al. (2003)
					Géminard et al. (2009)
GBP1 knockdown	Amino acids	No effect on DILP2 secretion (i.e., the protein does not accumulate in the IPCs)	DILP5 accumulates in the IPCs	Not known	Koyama and Mirth (2016)
GBP1&2 mutants and Knockdown	Protein/Amino acid	Accumulation of DILP2. *dilp2* mRNA levels elevated in GBP1/2 knockdown animals	Accumulation of DILP5. *dilp5* mRNA downregulated	Not known	Koyama and Mirth. (2016)

## 4 Neurons, nutrient sensing and metabolism

The nervous system plays an important role in integrating the process of nutrient sensing with regulation of metabolic status. However, our understanding of neuronal circuits associated with nutrient sensing, DILP secretion and metabolic regulation even in a relatively simple organism like *Drosophila* is still very limited. The role of different neurons and neuronal circuits involved in regulating IPC function and metabolism is discussed below:

### 4.1 Glutamatergic neurons and regulation of IPCs

Glutamatergic inputs to the IPCs emerge from a subset of interneurons in the medial ventral ganglion (mVG). These neurons, marked by VGN6341-GAL4 make direct synaptic contacts with a subset of DILP2 expressing IPCs. Activation of these neurons stimulates the IPCs and induces DILP secretion (Jayakumar et al., 2016). Under protein deficient conditions, the VGN6341 neurons receive cholinergic inputs from Pick-pocket (Ppk) positive multidendritic (MD) neurons eliciting Ca2+ transients at the mid- 3rd instar larval stage, essential for signaling to downstream peptidergic neurons including the IPCs, leading to release of DILP2 and eventually pupariation. As with the fat body, sensing of amino acids by Ppk neurons is mediated by amino acid transporter-Slimfast. Thus, the MD-VGN6341-IPC circuit appears to be activated under conditions of protein deprivation to promote animal survival by ensuring pupariation albeit delayed, with glutamatergic neurons being central to the circuit (Jayakumar et al., 2016; Jayakumar et al., 2018).

### 4.2 GABAergic and serotonergic regulation of IPCs

The IPCs express receptors for serotonin, GABA and octopamine suggesting regulation by neurons that express these neurotransmitters and neuromodulators (Nassel et al., 2013). Inputs by GABAergic neurons are inhibitory in nature, preventing activation of its downstream target. A subset of the GABAergic neurons in the region surrounding the IPCs express Domeless, the receptor for cytokine Upd2 which is released by the fat body under nutrient rich “fed” conditions. Activation of Domeless inhibits release of GABA, thus relieving the repression on IPCs in both larval and adult stages (Rajan and Perrimon., 2012).

These GABAergic neurons are thus part of the circuit that senses the “fed” state. The spatial proximity between these neurons and the IPCs suggests that they are likely to form synaptic connections although this needs to be confirmed.

Of the different serotonergic receptors, 5-HT1A appears to be the only receptor expressed in the IPCs. Curiously, expression of this receptor is turned on in a few IPCs towards the end of the 3rd instar larval stage in a few animals (upon cessation of feeding) but is detected in adult IPCs (Luo et al., 2012). The 5-HT1A receptor is inhibitory, and knockdown of this receptor in the IPCs increases DILP2 immunoflourescence in the IPCs (Luo et al., 2012). This suggests that a subset of serotonergic neurons negatively regulate DILP2 synthesis and/or secretion from the IPCs. Consistent with this, *5-HT1A* mutants or flies having reduced expression of the receptor in the IPCs, exhibit lower lipid levels upon starvation consistent with high energy expenditure (Luo et al., 2012).

Even though the subset of serotonergic neurons that signal *via* 5-HT1A are yet to be identified, it is clear that there are other subsets which are likely to influence IPC activity indirectly. For instance, Nucleostemmin-3 (Ns3) a conserved protein of the Ylqf related family of GTPases (YRG) shows enriched expressed in serotenergic neurons. Interestingly, *ns3* mutant adults are smaller in size and exhibit elevated levels of serotonin in the adult brain. Further, mutant IPCs show an increase in DILP2 but no significant increase in transcript levels (Kaplan et al., 2008). This latter observation is consistent with the idea of serotonin signaling modulating secretion of DILP2 (Kaplan et al., 2008; Luo et al., 2012).

### 4.3 Octopamine and regulation of dilp expression

Octopamine and its precursor tyramine are important neuromodulators in insects and regulate many aspects of insect physiology akin to the beta-adrenergic system in vertebrates. A growing body of work shows that octopaminergic-tyraminergic neurons (marked by *tdc2*-GAL4) regulate IPCs (Crocker et al., 2010; Luo et al., 2014). Furthermore, these neurons or at least a subset of them, form direct synaptic connections with the cell bodies of the IPCs (Dhiman et al., 2019).

Of the multiple octopamine receptors, the OAMB-K3 splice form is expressed in the IPCs (Crocker et al., 2010) and knockdown of OAMB in the IPCs increases expression of *dilp3* mRNA but not of *dilp 2,5* (Luo et al., 2014). Furthermore, the overall lipid levels in these flies is significantly decreased under fed and starved conditions (Luo et al., 2014). Interestingly, however, flies lacking tβh, the enzyme that synthesizes octopamine, exhibit elevated levels of triacylglycerol (TAG) indicating that loss of octopamine signaling (through OAMB-K3) promotes fat storage (Li et al., 2016).

**FIGURE 2:**
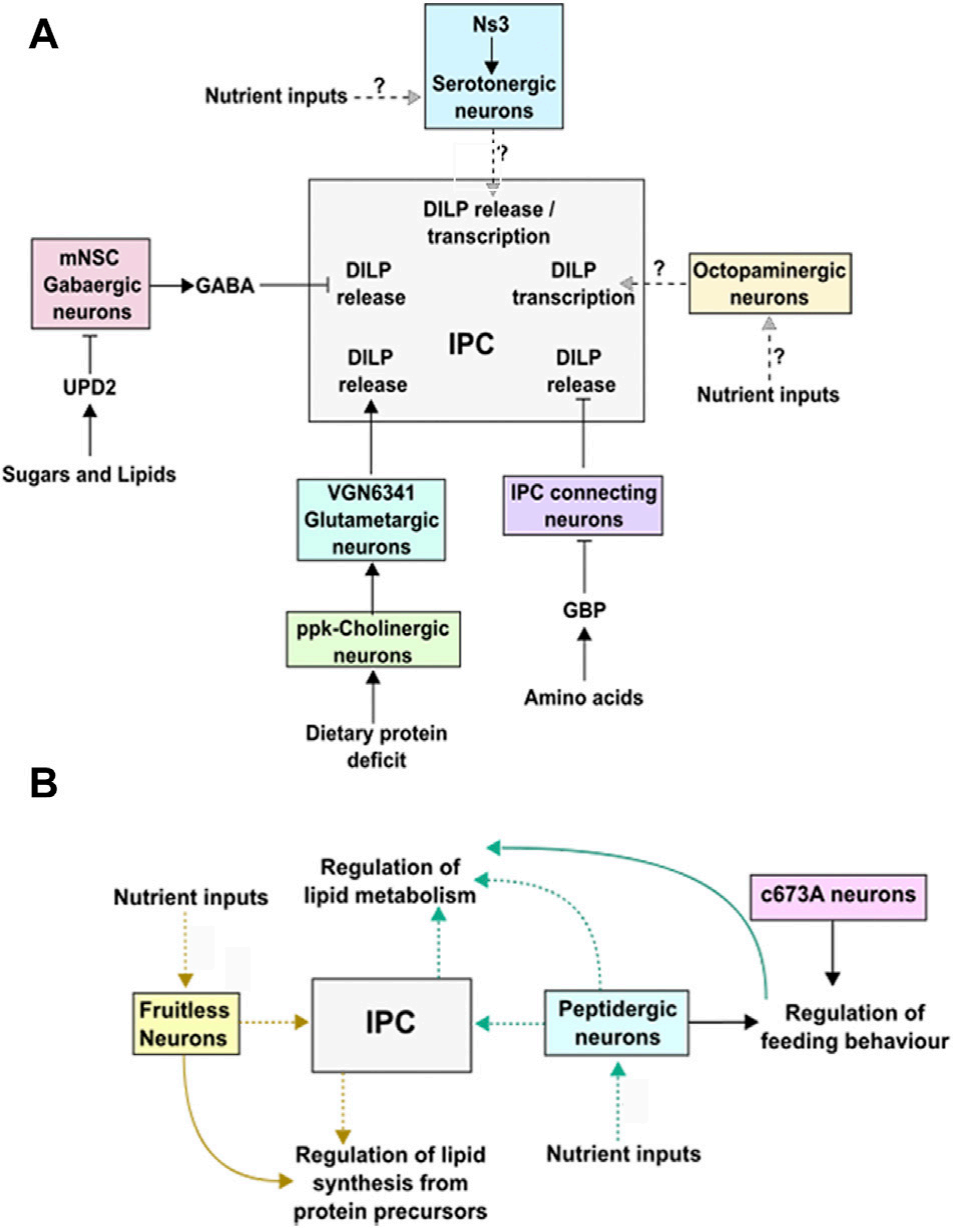
**(A)** A diagram showing the inputs from different neurons and their effect on *dilp* transcription and/or release. **(B)** Diagram showing neurons associated with lipid synthesis and metabolism. The dotted lines indicates pathways that need to be elucidated.

Despite the involvement of octopamine in a wide range of stress and non-stress related behaviors, their role in DILP regulation remains poorly understood. In this context it has been shown that Mon1, a component of the GEF complex for Rab7, functions in Tdc2 positive neurons to regulate *dilp* expression in the IPCs (Dhiman et al., 2019). Interestingly, adult *mon1* mutants are smaller in size and show reduced mRNA expression of multiple *dilps* including *dilps 3, 5*. Consistent with this, systemic insulin signaling is also affected in these mutants (Dhiman et al., 2019). Whether octopaminergic neurons are part of a larger nutrient sensing and metabolic circuit remains to be elucidated. A summary of the circuits and neurons involved in regulating DILP synthesis and release by the IPCs is shown in Figure 2A.

### 4.4 Neurons and lipid storage

While there is some understanding of neurons associated with nutrient sensing and insulin expression and secretion, less is known of neurons and circuits whose activity influences metabolism and energy storage independent of the IPCs. In *Drosophila*, neutral lipids, constitute an important source of energy which is stored in the fat body and oenocytes which are equivalent of the mammalian liver. The latter is also the site of *de novo* lipid synthesis.

Sensing lipids involves communication between the intestine, where lipids are absorbed, the fat body/oenocytes where lipids are stored, and the brain. Through an activity-based screen, Al-Anzi and colleagues have identified two sets of neurons associated with fat storage in *Drosophila* marked by c673a-GAL4 and fru-GAL4. While the two sets share a few neurons in common including the IPCs (median neurosecretory cells), the ability to regulate fat storage in each case is seen to arise from the activity of neurons that are non-overlapping between the two sets. Furthermore, the mechanisms leading to increase in fat storage is different: silencing activity in c673a-GAL4 leads to an increase in food consumption while in case of fru-GAL4 neurons, increase in lipids storage arises from changes in metabolism wherein protein precursors are shunted towards lipid synthesis (Figure 2B; Al-Anzi et al., 2009). Interestingly, knock-down of the IP3 receptor (*itpr*) in Dimmed positive peptidergic neurons also results in resistance to starvation accompanied by elevated levels of TAG, that is not observed upon knock-down of *itpr* in the IPCs alone. However, as in the case of c673a-GAL4, knock-down of *itpr* in peptidergic neurons leads to hyperphagia (Subramanian et al., 2013). Together, these studies point to non-IPC mediated regulation of fat metabolism and storage. Determining the physiological inputs that trigger activity of these neurons remains to be addressed.

## 5 Neurons, insulin-like peptides and ecdysone signaling

Ecdysone is a steroid hormone in insects which controls transitions between different developmental stages. The synthesis and release of ecdysone is tightly coupled to environmental conditions including nutrition to ensure survivability. The cross-talk between insulin signaling and ecdysone release involves a pair of neurons in the brain that express “Imaginal morphogenesis protein–Late2” or ImpL2 for short. ImpL2 produced in these neurons is essential for uptake of DILP2 to trigger insulin signaling in the brain-ring gland circuit to regulate release of ecdysone (Bader et al., 2013; Sarraf-Zadeh et al., 2013). Thus, ImpL2 neurons help co-ordinate growth and developmental timing to nutritional status. A very recent study has now shown that ImpL2 expressed by corpora-cardiaca (CC) helps “store” DILP2/5 to make them available under conditions of starvation that the animal in the late larval stage might encounter post the nutrient restricting checkpoint (NRC). Under these conditions, DILPS stored in the CC activate insulin signaling in the prothoracic gland to trigger ecdysone release essential for pupariation (Ghosh et al., 2022). The ImpL2 neurons thus serve as an example of neurons associated with restricting insulin signaling along the brain-ring gland axis. It is conceivable that other such neurons, some specific to other DILPs, exist to regulate brain-organ signaling.

At the systemic level, ImpL2 released by organs including the fat body and muscle, function to dampen sensitivity to circulating insulin to help animals to cope with starvation (Honegger et al., 2008).

## 6 Concluding remarks and open questions

In *Drosophila*, as in vertebrates, the brain is integral to mechanisms involved in nutrient sensing and metabolism. The IPCs which are central to DILP production are regulated by inputs from different neurons. The circuits formed by these neurons to link peripheral “sensing” organs (fatbody, muscle) to the “central integrator” (IPCs) are still largely unknown.

Of the inputs received by the IPCs, the serotonergic and GABAergic neurons are inhibitory, while the glutamatergic inputs stimulate IPCs in response to amino acids sensed by cholinergic neurons in the periphery. The GABAergic neurons function as part of a circuit that senses the “fed” state. However, less is known about the regulation by serotonergic neurons: A subset of these that activate 5HT-1A in the IPCs are inhibitory (Luo et al., 2012). However, the study on ns3 (neurostemmin3) suggests that another subset of these neurons are likely to be positive regulators of growth. Whether they constitute a part of a nutrient sensing circuit is still unclear.

Another important class of neurons that regulate IPCs are the Octopaminergic/tyraminergic neurons which are analogous to the norepirephrine/epinephrine (aka nor-adrenalin/adrenalin) system in vertebrates. They co-ordinate the response of organs to various internal and external stresses through altered physiology, changes in metabolism or behaviour. In the context of nutrition, these neurons regulate feeding and also modulate response to starvation. Despite the wide impact that these neurons have on the well-being and fitness of the animal, little is known of whether these neurons are associated with nutrient sensing. In this context, Dhiman et al. (2019) show that Monensin Sensitivity 1 (Mon1) in octopaminergic/tyraminergic (OPN) neurons regulates expression of *dilps* in the IPCs. Consistent with its role in regulating insulin signaling, homozygous *mon1* escaper mutants have a short life span, are smaller in size and are sterile. Given the role of the endo-lysosomal pathway in cellular nutrient sensing, and the large number of physiological processes regulated by the OPNs, it would be interesting to test if these neurons are associated with “nutrient sensing” and growth.

From studies carried out thus far it is evident that nutrient sensing is a complex process and the response of the IPCs to changes in activity of different neurons is just as complex and our comprehension constitutes just the tip of the iceberg. Identifying precisely, the neurons that relay information on specific nutrients to the IPCs will help dissect and improve our understanding of the process.
